# Iron regulatory protein (IRP)-iron responsive element (IRE) signaling pathway in human neurodegenerative diseases

**DOI:** 10.1186/s13024-017-0218-4

**Published:** 2017-10-23

**Authors:** Zhi Dong Zhou, Eng-King Tan

**Affiliations:** 1 National Neuroscience Institute of Singapore, 11 Jalan Tan Tock Seng, Singapore, 308433 Singapore; 2 Department of Neurology, Singapore General Hospital, Outram Road, Singapore, 169608 Singapore; 3 Signature Research Program in Neuroscience and Behavioral Disorders, Duke-NUS Graduate Medical School Singapore, 8 College Road, Singapore, 169857 Singapore

**Keywords:** Amyloid precursor protein, α-synuclein, Iron, Iron-responsive element, Iron-binding proteins, Human neurodegenerative diseases

## Abstract

The homeostasis of iron is vital to human health, and iron dyshomeostasis can lead to various disorders. Iron homeostasis is maintained by iron regulatory proteins (IRP1 and IRP2) and the iron-responsive element (IRE) signaling pathway. IRPs can bind to RNA stem-loops containing an IRE in the untranslated region (UTR) to manipulate translation of target mRNA. However, iron can bind to IRPs, leading to the dissociation of IRPs from the IRE and altered translation of target transcripts. Recently an IRE is found in the 5′-UTR of amyloid precursor protein (APP) and α-synuclein (α-Syn) transcripts. The levels of α-Syn, APP and amyloid β-peptide (Aβ) as well as protein aggregation can be down-regulated by IRPs but are up-regulated in the presence of iron accumulation. Therefore, inhibition of the IRE-modulated expression of APP and α-Syn or chelation of iron in patient’s brains has therapeutic significance to human neurodegenerative diseases. Currently, new pre-drug IRE inhibitors with therapeutic effects have been identified and are at different stages of clinical trials for human neurodegenerative diseases. Although some promising drug candidates of chemical IRE inhibitors and iron-chelating agents have been identified and are being validated in clinical trials for neurodegenerative diseases, future studies are expected to further establish the clinical efficacy and safety of IRE inhibitors and iron-chelating agents in patients with neurodegenerative diseases.

## Background

The roles of iron in hemoglobin formation and oxygen transport have been linked to human health and diseases [1]. Iron is important to the functioning of many prosthetic groups, including haem and iron-sulphur clusters, and iron depletion can contribute to anemia [2]. However, excess iron can promote the generation of deleterious reactive oxygen species (ROS) and is linked to both haemochromatosis and thalassaemia [3, 4]. The molecular mechanisms of iron metabolism in humans have been extensively studied. Ferrous iron that is absorbed from intestinal lumen into enterocytes can be exported into bloodstream via ferroportin (Fpn), inhibited by hepcidin or facilitated by hephaestin and ceruloplasmin (CP) with ferroxidase activities [5–7]. The iron in bloodstream can be captured by transferrin (Tf) in ferric state and transported to peripheral tissues [8]. In peripheral tissues the iron loaded Tf will be recognized by transferrin receptor (TfR) on cell membrane, followed by receptor-mediated endocytosis [8]. In acidic endosome the iron will be dissociated from Tf and released into cytoplasm via divalent metal transporter 1 (DMT1) after reduction to its ferrous state by a STEAP family reductase [8]. In the cytoplasm, free ferrous iron can be immediately used as a co-factor for enzyme such as tyrosine hydroxylase or taken up by mitochondria via mitoferrin for synthesis of Fe-S clusters and heme groups, which is indispensible for mitochondria functions [8, 9]. Considerable amount of iron in cells can be sequestered and stored in cytosol ferritin or mitochondrial ferritin (MtFt) in ferric state [9, 10]. Excess iron can be exported into bloodstream through Fpn to form ferric iron-Tf complex again for iron re-distribution [11].

A substantial amount of iron can be absorbed into brain, mainly in the substantia nigra pars compacta (SN) [12–14]. The iron in bloodstream can be transported across blood brain barrier (BBB) through brain capillary endothelial cells (BCECs) via Tf-TfR and DMT1-Fpn pathways [15]. The circulating iron-Tf complex can be captured by TfR on BCECs cell membrane, internalized via endocytosis, released to BCECs cytoplasm via DMT1 and exported into brain interstitial fluid via Fpn [15]. In brain iron plays multiple physiological roles including neurotransmitter synthesis, neuron myelination, mitochondrial functions and energy generation [15]. Iron homeostasis in the brain is precisely controlled and dysregulated brain iron homeostasis (iron overload or deficiency) can lead to brain disorders [15]. Brain iron deficiency (BID) can disturb brain development and functions [16]. BID can be associated with the pathogenesis of brain disorders including Attention Deficit Hyperactivity Disorder (ADHD) and Restless Legs Syndrome (RLS) [17–21]. The iron overload in brain can also be a pathological factor for brain disorders, including Alzheimer’s disease (AD) [22, 23], Parkinson’s disease (PD) [23, 24], and other human brain disorders [25–30].

## Main text

### Modulation of iron homeostasis by IRP-IRE signaling pathway

**Fig. 1:**
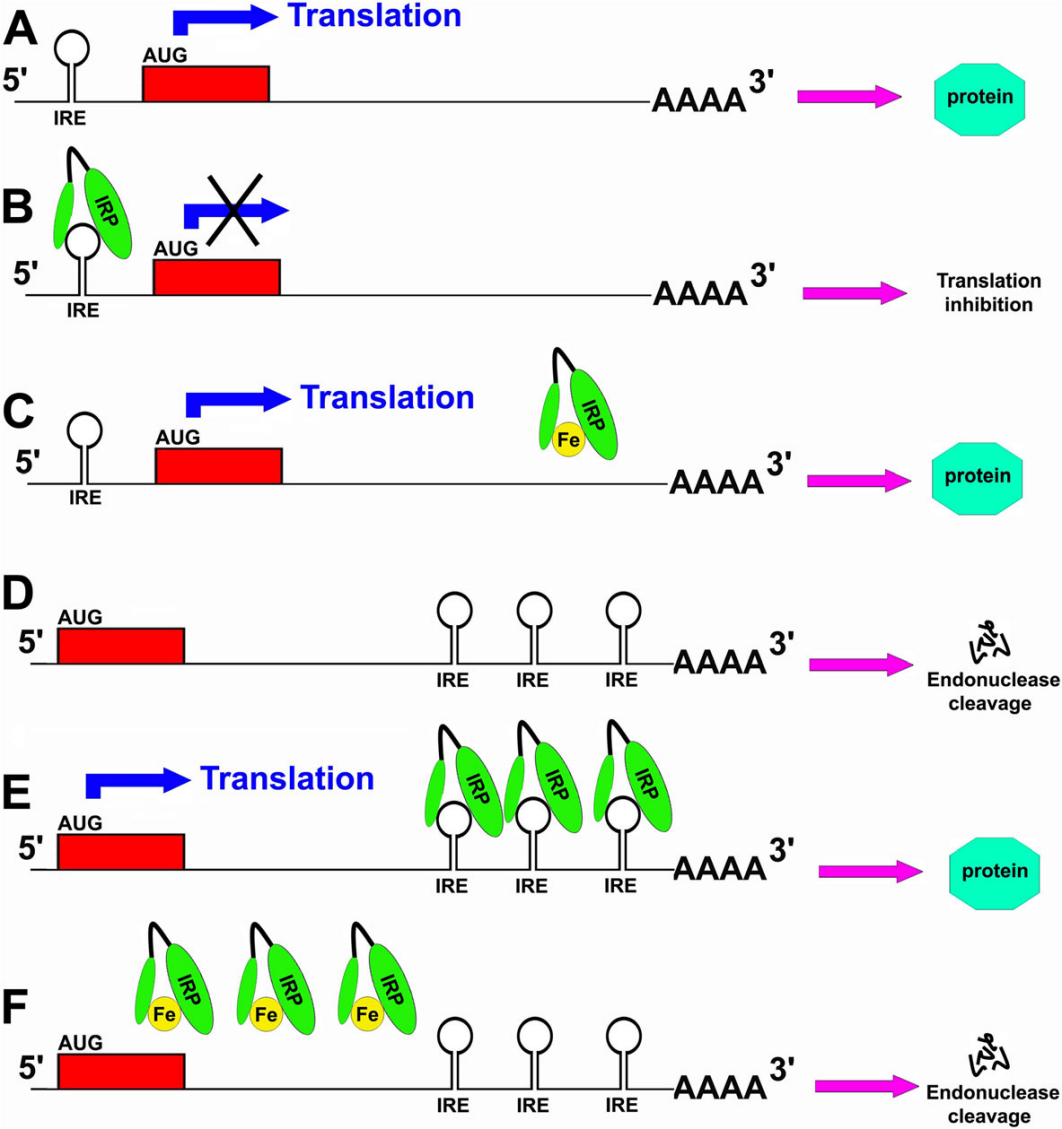
Mechanism of translation modulation by the IRP-IRE signaling pathway. **a** The translation of transcripts containing an IRE in the 5′-UTR can occur in the absence of IRP binding to the IRE; (**b**) after the binding of IRP to the IRE in the 5′-UTR, translation of the transcripts is inhibited. **c** Iron can bind to IRP to induce a transformational change of IRP, leading to its dissociation from the IRE, which can promote the translation of transcripts. **d** The IRE can be present in the 3′-UTR of the transcript. Without IRP binding to the 3′-UTR, the transcript can be susceptible to endonuclease attack and degradation, leading to down-regulation of translation. **e** Binding of IRP to the IRE in the 3′-UTR can protect transcripts against endonuclease degradation and therefore promote the translation of transcripts. **f** Iron can bind to IRP, leading to the dissociation of IRP from the IRE in the 3′-UTR, endonuclease attack, and the degradation of transcripts, which down-regulates the translation of transcripts

Iron homeostasis is elaborately regulated [8]. Although some control mechanisms exist at the transcriptional level, the absorption, transportation and storage of iron are meticulously modulated at the translational level by the iron regulatory protein (IRP) and iron-responsive element (IRE) signaling pathway [31–33]. IRP1 (90 KDa) and IRP2 (105 KDa) are two known RNA-binding proteins, and their inducible interactions with IRE function to control the translation of ferritin and Fpn mRNA and the stability of TfR mRNA. Briefly, the IRPs can control iron metabolism via binding to specific non-coding sequences, known as IREs, within the untranslated region (UTR) of mRNA transcripts [32]. The IREs are 30-nucleotide long RNA motifs containing the CAGUGN sequence (the classic IRE motif) and can form special stem-loop structures [32, 34]. IREs can be present in either the 3′-UTR or 5′-UTR of the target mRNA [32, 34]. Transcripts with IRE motifs in their 5′-UTR include the ferritin H and L subunits, Fpn and aminolevulinic acid synthetase [8, 35], whereas target mRNA with IRE motifs in the 3′-UTR include TfR (5 copies) and DMT-1 [35]. The detailed IRP-IRE signaling pathway translation modulation mechanisms are illustrated in Fig. 1.

IRPs can act as either a translational enhancer or a translational inhibitor [32, 34]. In iron-deficient cells, the interaction between IRP and the IRE motif in the 5′-UTR of target mRNA can abrogate translation via interruption of important interactions between the mRNA and ribosome for the initiation of translation [32]. However, in iron-replete cells, iron can bind with IRPs to induce a conformational change, which promotes the dissociation of IRPs from the target mRNA, leading to facilitation of the translation of the target mRNA [32]. In contrast, some mRNA transcripts contain one or more IRE motifs in their 3′-UTR. In iron-deficient cells, the binding of IRPs to an IRE at the 3′-UTR of transcripts can protect target mRNA against endonuclease cleavage [32]. Therefore, the interaction between IRPs and a 3′-UTR IRE can extend the half-life of transcripts and promote translation of target mRNA. However, in iron-replete cells, the dissociation of IRP from an IRE at the 3′-UTR renders target transcripts susceptible to endonuclease attack and degradation, leading to down-regulation of the translation of transcripts [32]. Both transcripts of ferritin and Fpn have IRE in their 5′-UTRs, so that under iron deficiency condition the translation of ferritin and Fpn can be inhibited by IRPs [8]. The decreased expression of ferritin and Fpn can reduce the unnecessary iron binding by ferritin and iron export by Fpn, leading to an increased level of free iron available for cell usage. However both TfR and DMT1 mRNA have 3′-UTR IREs, which can bind to IRPs under iron deficiency, leading to stabilization of transcripts and subsequent increased synthesis of TfR and DMT1 to promote iron absorption into cells [8]. In contrast, under a situation of iron accumulation, the increased iron level can disturb the IRP-IRE interaction to promote the translation of the ferritin and Fpn transcripts as well as to destabilize TfR and DMT1 mRNA [32]. Therefore under iron accumulation, the iron absorption will be inhibited, while iron storage and export can be enhanced. [8]. Pathological factors inducing a disturbance of the IRP-IRE signaling pathway will impair iron homeostasis, which can contribute to the onset and development of human disorders. However, one recent study reported a novel mechanism for the iron-induced modulation of translation of target mRNA [36]. They demonstrated that eukaryotic initiation factor 4F (eIF4F) can specifically bind to an IRE at the 5′-UTR of target mRNA with high affinity, which is vital for translation initiation [36]. However, iron can also directly bind to the IRE of target mRNA, leading to a conformational alteration of mRNA [36]. The conformational changes in mRNA induced by the binding of iron will facilitate the interaction between eIF4F and IRE-RNA, which can outcompete binding between IRP and the IRE [36]. Therefore, accumulated iron can contribute to up-regulated translation of target mRNA with a 5′-UTR IRE [36]. Recent evidence suggests that the IRP-IRE signaling pathway may play other physiological roles beyond iron homeostasis. Functional IREs are identified in both transcripts of CDC14A (cell division cycle 14 homologue A) and hypoxia-inducible factor 2a (HIF2a) [37–39]. The translation of CDC14A, a phosphatase involved in cell cycle, can be modulated by IRP-IRE signaling [37, 38]. The iron depletion can lead to growth inhibition at the G1–S transition [40]. These findings indicate other important physiological roles of the IRP-IRE signaling pathway beyond maintenance of iron homeostasis.

### Linking IRP-IRE signaling pathway to human neurodegenerative disorders

However, recent findings indicate that the iron accumulation-induced dysfunction of the IRP-IRE signaling pathway may contribute significantly to protein aggregation and neuron loss in AD and PD, which sheds new light on the pathogenesis and therapy of the neurodegeneration observed in AD and PD.

AD is the most common form of dementia in the elderly [41, 42]. The pathology of AD is characterized by synaptic defects, neuron loss and the formation of amyloid β-peptide (Aβ) plaques in the hippocampus and cortical areas [41, 42]. Aβ plaques contain Aβ aggregates and neurofibrillary tangles formed by aggregated forms of the microtubule-associated protein tau [41, 42]. Aβ is generated during protease cleavage of amyloid precursor protein (APP) and is critical in AD pathogenesis [43]. APP is a single transmembrane metalloprotein that can be cleaved via two main proteolytic procedures: amyloidogenic processing and non-amyloidogenic processing [43]. The amyloidogenic processing of APP can generate Aβ peptides 40–42 amino acids in length. Among the Aβ peptides produced, Aβ42 is more apt to form fibrillar protein aggregates [43]. Up-regulated APP expression and Aβ levels are associated with neuronal loss in AD [43]. Furthermore, increased levels of APP can also be linked to amyloidosis in Down syndrome (DS), since the APP gene is located on chromosome 21, which is triplicated in DS [44, 45]. The increased level of APP can be a pathological factor and a therapeutic target linked to Aβ amyloidosis and neuron loss in AD and DS.

However, recent findings suggest that the increased APP level, aggravated Aβ deposition and neuron loss in AD can be induced by iron accumulation and disturbance of the IRP-IRE signaling pathway [46, 47]. A novel functional IRE is found in the 5′-UTR of the APP mRNA transcript (+51 to +94 from the 5′-cap site) [48–50]. The IRE in APP mRNA is located immediately upstream of an interleukin-1 responsive acute box domain (+101 to +146) [48–50]. The IRE in APP mRNA can bind specifically to IRP, which can be abrogated by a mutation in the core IRE motif [50]. In the presence of iron chelators, the translation of APP mRNA can be selectively down-regulated [48, 51]. In contrast, iron influx or an increased iron level can enhance APP mRNA translation and Aβ generation [48, 51]. Therefore, these findings demonstrate that iron accumulation can be a pathological factor triggering the up-regulated expression of the APP holoprotein and subsequent Aβ deposition. However, therapeutic strategies targeting the IRE in APP mRNA should alleviate Aβ deposition and neuron loss in AD brains. The strategy of targeting the APP 5′-UTR to reduce APP expression and Aβ amyloid formation has been validated by the usage of novel IRE chemical inhibitors to alleviate APP and amyloid levels as well as cognitive decline in the TgCRND8 AD mouse model [52]. Identified potent IRE inhibitors, such as Posiphen, have been reported to be bona fide APP 5′-UTR-directed translation blockers that can reduce Aβ generation in the cerebrospinal fluid (CSF) of humans and have entered clinical trials for AD therapy [53]. In contrast, iron species can be a factor promoting ROS generation and inducing Aβ aggregation [54]. The increased iron level in AD patient brains is found to be relevant to the formation of neurofibrillary tangles [15]. The therapeutic strategies using iron chelators plus IRE inhibitors may act collaboratively to alleviate Aβ aggregation and rescue neuron loss in AD patient brains.

Parkinson’s disease (PD) is the second most common neurodegenerative disorder, and the estimated incidence of PD is approximately 1% of the population over the age of 55 [55, 56]. PD is characterized by progressive DA neuron loss in the SN as well as Lewy body formation in affected brain areas [55, 56]. The pathogenesis of the progressive DA neuron degeneration in PD is still unclear as of yet, and no effective therapy has been developed to alleviate the progressive DA neuron degeneration in PD. However, recent new findings on the roles of the IRP-IRE signaling pathway in PD add to the pathogenesis and therapy of iron-relevant DA neuron degeneration in PD. The involvement of IRPs in DA neuron degeneration can be supported by 2 in vivo studies on IRP2 knockout mice models [57, 58]. In middle to late age (18 to 24 months), mice lacking IRP2 will develop abnormal motoric PD-like behaviors including tremors at rest, abnormal gait, and bradykinesia [57, 58]. Furthermore, significant iron accumulation in the brains of IRP2 knockout mice precede the onset of DA neuron degeneration and PD-like symptoms by many months, accompanied by the deposition of ubiquitin-positive protein aggregates and inclusions in the mouse brain [58]. The DA level in the dorsal striatum of IRP2 knockout mice decreased (approximately 20%) significantly compared with that of control mice, suggesting that IRP2 knockout induced DA neuron degeneration in the mouse brain [57]. Previous findings demonstrated that iron retention and iron dependent toxicity to DA neurons can be induced by nitric oxide (NO) via facilitation of interactions between IRP and IRE to inhibit translation of ferritin, Fpn and other iron metabolism relevant proteins [59, 60]. Furthermore the NO can inhibit APP translation to aggravate the iron retention in DA neurons, as APP stabilizes Fpn to promote iron export [61]. NO is supposed to be a causative factor upstream of nigral iron accumulation and DA neuron degeneration in PD pathogenesis [61]. These findings suggest a close association of iron and the IRP-IRE signaling pathway with DA neurodegeneration in PD.

**Fig. 2:**
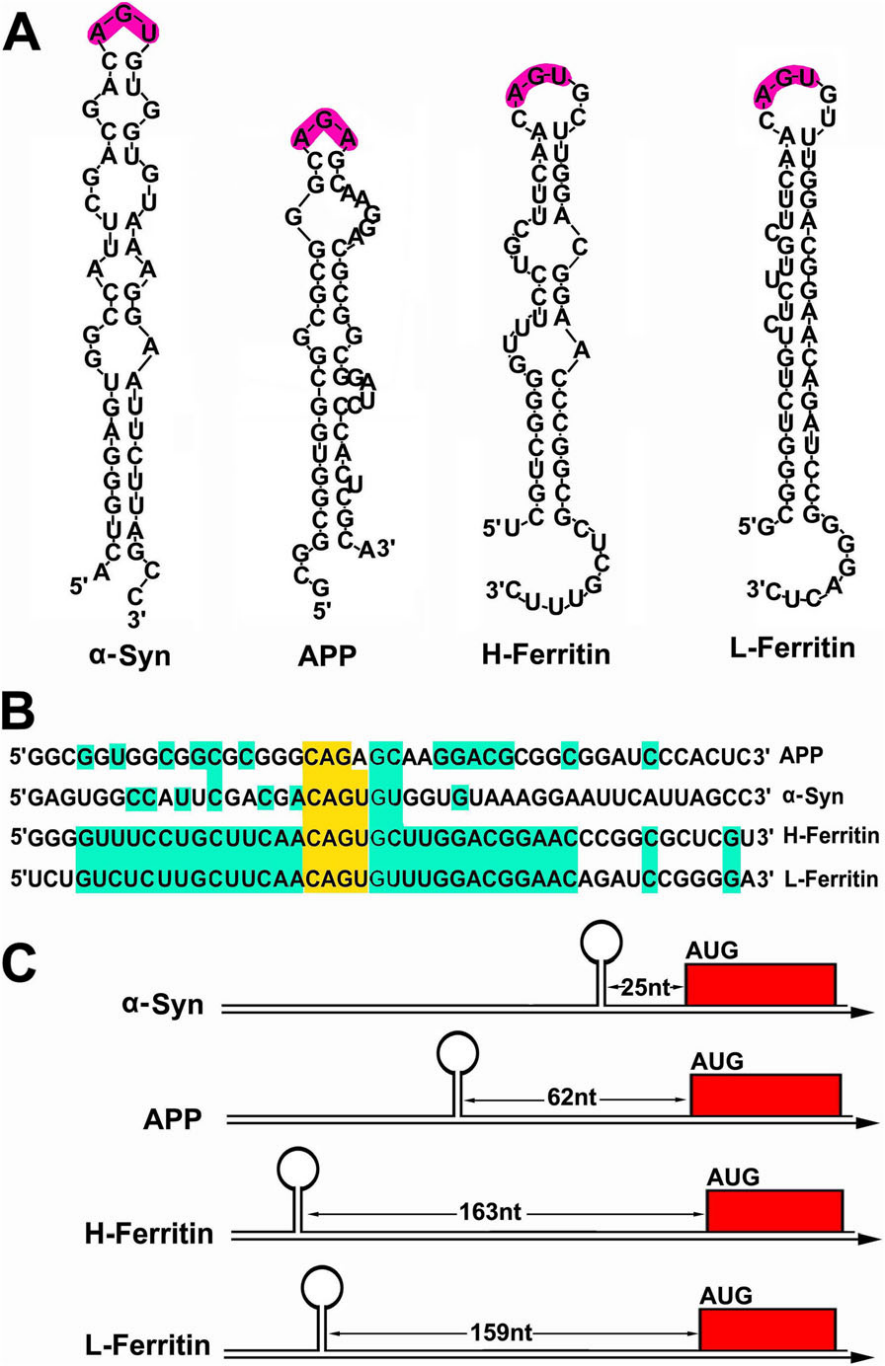
Comparison of the 5′-UTR IRE stem-loop of the α-Syn, APP, H-ferritin and L-ferritin transcripts. **a** Stem-loop structures of α-Syn, APP, H-ferritin and L-ferritin. **b** Relative alignment of the sequences encoding the 5′-UTR-specific IRE stem-loops in α-Syn, APP, H-ferritin and L-ferritin. The key IRE motifs of AGU or AGA are highlighted in purple. B. Homological analysis of the IRE stem-loop sequences of the α-Syn, APP, H-ferritin and L-ferritin transcripts. Key IRE motifs are highlighted in yellow, while homological nucleotides in the flank areas of the key IRE motifs are highlighted in cyan. **c** Maps of the 5′-UTR IRE stem-loops in the α-Syn, APP, H-ferritin and L-ferritin transcripts

Furthermore previous evidence suggests that iron accumulation and dysfunction of the IRP-IRE signaling pathway can be linked to α-Syn-induced toxicity of DA neurons [62, 63]. α-Syn is the central protein involved in the pathogenesis and therapy of PD [64, 65]. Lewy bodies consisting of aggregated α-Syn can be found in almost all PD patient brains [66, 67]. α-Syn mutations are known to be linked to the onset of the familial form of PD (FPD) [68–70]. Furthermore, the increased level of the α-Syn protein in the brain due to gene duplication or triplication is also linked to the onset of FPD [71, 72]. However, human post-mortem brain studies have demonstrated that while the mRNA levels of α-Syn in PD brains are either unchanged or decreased, higher levels of the insoluble α-Syn protein occur during PD progression [62]. In contrast, disrupted iron metabolism in the SN is a common feature of PD [46]. Studies have shown that the iron levels can be up-regulated to almost 2-fold in a single SN DA neuron in PD patient brains [24]. The elevated iron levels in PD patient brains are thought to be linked to the accelerated deleterious formation of α-Syn protein aggregates [63]. Furthermore, novel evidence has shown that elevated iron levels in the brain can cause enhanced α-Syn protein translation in the brain [49]. A unique RNA stem-loop with an IRE motif (CAGUGN) is found in the 5′-UTR of α-Syn mRNA [49, 62, 73]. The IRE stem-loop of the α-Syn transcript spans two exons [73]. IRPs can bind with the IRE of α-Syn mRNA and inhibit translation of the α-Syn transcript [49]. However, iron can reverse the translation inhibition of α-Syn by IRPs and therefore facilitate α-Syn expression and aggregation [49]. The translation of α-Syn mRNA can be inhibited by new IRE chemical inhibitors [73, 74]. These findings suggest a pathological relevance of iron and the IRP-IRE signaling pathway in the α-Syn-dependent toxicity of DA neurons in PD. Iron chelators and new IRE inhibitors should alleviate iron-dependent α-Syn toxicity and DA neuron degeneration in PD [73, 75, 76]. The detailed IRE sequences and stem-loop structures as well as maps of the IRE stem-loop in α-Syn and APP transcripts are illustrated in Fig. 2.

The iron and IRP-IRE signaling pathway also seem to be implicated in the pathogenesis of ALS [77, 78]. ALS is a progressive neurodegenerative disorder induced by multiple pathogenic factors and is characterized by the progressive and selective loss of motor neurons in the cerebral cortex, brainstem, and spinal cord [77]. α-Syn-positive inclusion bodies can be identified in ALS [79, 80]. Increasing evidence indicates a pathological role of iron dysregulation in neuronal cell death in ALS [77, 81–83]. The role of iron in the pathogenesis of ALS is supported by the therapeutic effects of an iron chelation strategy in ALS mouse models [84–86]. Furthermore, a potential IRE has been computer-predicted to exist in the 5′-UTR of mRNA encoding C9orf72, a key ALS-linked gene [87]. This RNA structure is proposed to post-transcriptionally regulate the expression of the C9orf72 protein in response to iron dyshomeostasis [87]. Such a hypothesis can be supported by previous findings on the increased levels of iron and ferritin in patients with ALS [87]. Thus, a potential mechanistic link may be present between iron and the iron-modulated translation of C9orf72, which is associated with ALS pathogenesis [87].

### Linking iron and the IRP-IRE signaling pathway to other human disorders

Although none of the point mutations in IRP1 or IRP2 have been confirmed to cause human disease, some preliminary studies have implicated the potential association of SNPs or mutations of IRPs with human diseases. A genome-wide association study (GWAS) identified three IRP2 SNPs associated with patients with chronic obstructive pulmonary disease (COPD) [88]. The levels of IRP2 protein and mRNA have been found to be higher in lung tissue samples from COPD patients than from controls [88]. These findings have indicated that IRP2 is a COPD-susceptible gene. Further studies are needed to establish the potential pathogenic role for IRP2 in COPD.

Mutations in the IRE motif have been identified in other human disorders beyond human neurodegenerative diseases [89–95]. Multiple point mutations in the IRE motif of the L-ferritin transcript have been linked to hyperferritinemia-cataract syndrome (HHCS) with hyperferritinemia and autosomal dominant congenital cataract [89–91]. The disease is characterized by early-onset, bilateral nuclear cataracts and up-regulated levels of serum ferritin [93]. However, HHCS patients demonstrate no hematological or biochemical abnormalities in iron metabolism [89, 90]. The serum iron level and transferrin saturation of HHCS patients are normal or low [89, 90]. The level of body iron is also not increased, therefore excluding iron overload as the pathogenic cause of HHCS [89, 90]. Studies have demonstrated that the point mutation in the IRE motif of L-ferritin mRNA disturbs the IRP interactions of the highly conserved IRE stem-loop [89, 90]. Furthermore, the disease-relevant point mutations in the IRE motif of L-ferritin lead to the abolishment of the binding of IRPs to the L-ferritin transcript, resulting in significantly increased synthesis of L-ferritin in cells from HHCS patients, which is in agreement with the up-regulated serum ferritin levels observed in these patients [93]. The point mutation in the IRE of L-ferritin mRNA induces hyperferritinemia and may lead to the accumulation and aggregation of L-ferritin in the lens and the early onset of cataract. Furthermore, mutations in the IRE motif of the H-ferritin gene may also be relevant to human disease [92]. A point mutation (A49U) in the 5′-UTR IRE motif of the human H-ferritin gene has been identified in members of a Japanese family with dominantly inherited iron overload [92]. This point mutation in the IRE motif of H-ferritin increases its affinity for IRP binding, leading to suppression of H-ferritin synthesis, an increase in iron uptake, autosomal dominant iron overload and tissue iron deposition [92]. In addition to mutation-induced alterations in H- and L-ferritin, mutation-induced alterations in the expression of other IRE-modulated protein can also be disease-relevant. Mutations in the 5′-UTR downstream of the IRE motif of Fpn mRNA have been detected in a patient affected by hemochromatosis with iron overload [94]. Fpn is the major and sole iron exporter that transports ion out of cells [95]. The mutation in the 5′-UTR of the Fpn transcript may alter the IRE-IRP interaction, inhibit translation of Fpn, impair the exportation of iron from cells, and induce iron overload.

On the other hand, mutations in the coding areas of IRE-modulated genes have also been found to be relevant to human disease. The mutations of ferritin that lead to the impaired capacity of ferritin to retain iron within its iron core have been linked to PD as well as neuroferritinopathy, a severe dominantly inherited movement disorder characterized by the deposition of iron and ferritin in the brain, normal or low serum ferritin levels, and highly variable clinical features [96–98]. Mutations at the C-terminus of L-ferritin impair its stability and decrease its capacity to interact with iron, validated by the transgenic mouse model expressing mutant L-ferritin [99–101]. Other disease-relevant coding region mutations have been reported in other IRE-encoded genes, including erythroid 5-aminolevulinate synthase (eALAS, linked to sideroblastic anemia) [102–104], Fpn (hereditary hemochromatosis) [105, 106] and DMT-1 [107–109].

### Potential novel therapies using IRE inhibitors for human neurodegenerative diseases

The IRP-IRE signaling pathway has been implicated in the modulation of APP and α-Syn translation, which is important to neurodegeneration in PD and AD. Therefore, the identification of small molecular IRE chemical inhibitors to reduce APP and α-Syn levels and alleviate protein aggregation can have therapeutic significance to human neurodegenerative diseases [73, 110]. In principle, identified chemical IRE inhibitors can decrease ferritin and Tf expression to alleviate the excess iron accumulation in AD or PD brains. Furthermore, therapeutic IRE inhibitors that down-regulate APP and α-Syn protein translation and inhibit protein aggregation can promote neuronal survival. Therefore, potent non-toxic pre-drug IRE inhibitors with excellent BBB penetrating capacity should have high therapeutic significance in neurodegenerative diseases. So far, some promising drug candidates of IRE inhibitors have been identified and are being tested in different human clinical trials for AD and PD.

In one recent study, thirteen potent APP translation blockers that act selectively towards the uniquely configured IRE RNA stem-loop in the 5′-UTR of APP mRNA were identified from 110,000 compounds of a chemical library at Harvard [110]. Some of these chemicals were able to selectively reduce neural APP production in SH-SY5Y cells without affecting cell viability or the levels of α-Syn and ferritin [110]. In this study, the identified APP blocker-9 (JTR-009), a benzimidazole, was found to be superior to the other APP blockers in its ability to reduce the production of toxic Aβ in SH-SY5Y neuronal cells [110]. JTR-009 is thoughts to directly interact with the IRE in the 5′-UTR of APP mRNA and constitutively repress APP translation [110]. Furthermore, pifithrin-α (PFTα), an anti-stroke agent and a p53 inhibitor, was also found to have selective APP translation inhibition capacity [110]. However, JTR-009 was able to selectively inhibit APP expression, while PFTα could cause a dose-dependent down-regulation of APP, α-Syn and actin proteins in cells [110]. Furthermore, the potency of JTR-009 to inhibit the APP 5′-UTR-conferred translation was greater than that of posiphen ((+)-phenserine), a well-recognized and tolerated IRE inhibitor with both APP and α-Syn translation inhibitory capacity [110].

Posiphen is a small molecule drug derived from Calabar beans and is a phenyl carbamoyl analogue of (+)-physostigmine [73]. Identified from a nature product (NP) chemical library, posiphen is found to inhibit both APP and α-Syn protein translation [73]. Furthermore, the inhibitory effects of posiphen are potent and not toxic [73]. However, phenserine, its enantiomer with cholinesterase inhibitory capacity, has less inhibitory potency than posiphen as well as minor toxicity [73]. Another study demonstrated that posiphen and its metabolites, as well as phenserine, possess neuroprotective / neurotrophic capacities at concentrations of clinical relevance [111]. All compounds are found to potently inhibit the protein translation of APP and α-Syn in neuronal cells [111]. Therefore, posiphen and its metabolites, as well as phenserine, may be drug candidates for AD and PD. The posiphen-induced inhibition of IRE has been validated by various in vivo and in vitro studies [74, 112–114]. Recent phase 1 human clinical trials and a proof-of-concept study in subjects with mild cognitive impairment (MCI) have demonstrated the brain entry and capacity of posiphen to lower APP production in subjects, as assessed in CSF, by as much as 50% [115]. Currently phenserine is under phase 3 clinical trials for AD, while posiphen is in a phase 2 human clinical trial for AD and PD [116].

**Fig. 3:**
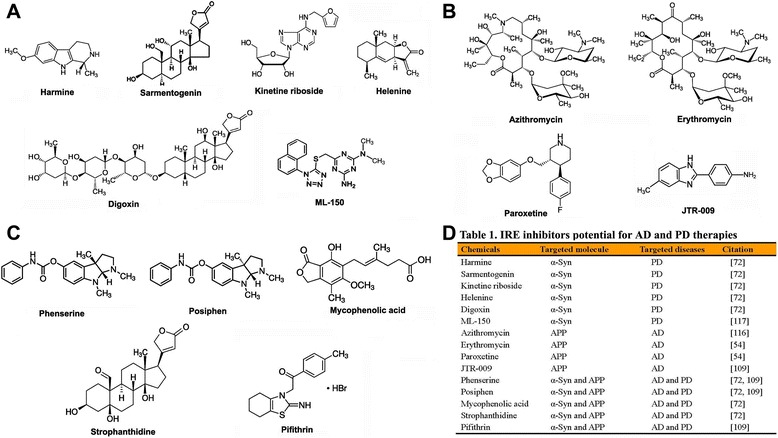
Identified small molecular IRE inhibitors that modulate the translation of α-Syn and APP. **a** Identified IRE inhibitors with a capacity to inhibit α-Syn translation. **b** Identified IRE inhibitors with a capacity to inhibit APP translation. **c** Identified IRE inhibitors with a capacity to inhibit both α-Syn and APP translation. **d** Table 1. Summary of IRE inhibitors potential for AD and PD therapies

Other potential chemical IRE inhibitors have also been reported. In a pilot study, paroxetine (a serotonin reuptake blocker), N-acetyl cysteine (anti-oxidant and Fe^2+^ chelator, NAC), and erythromycin (macrolide antibiotic) were employed to assess their anti-amyloid capacity in the transgenic TgCRND8 AD mouse model [52]. The levels of Aβ peptide were found to be reduced after the exposure of the mice to paroxetine, NAC, and erythromycin [52]. Other studies have verified that paroxetine can modulate APP expression via its actions on the 5′-UTR of the APP transcript [116, 117]. Therefore, paroxetine may be a chemical IRE inhibitor that can modulate APP expression. Another high-throughput screening study to search for novel, nontoxic, and selective small-molecule inhibitors of α-Syn expression has been performed [118]. From 303,224 screened compounds, one compound (ML150) displayed very potent inhibition of α-Syn expression [118]. ML150 specifically reduced α-Syn translation and likely acts via an interaction with and inhibition of the IRE of the α-Syn transcript [118]. These findings suggest that ML150 is a novel IRE inhibitor that specifically modulates α-Syn expression. The detailed molecular structures of the identified IRE chemical inhibitors as well as their potential relevance to therapies against human neuron degenerative diseases are illustrated and summarized in Fig. 3.

### The pathological and therapeutic targets in the iron-IRP-IRE signaling pathway for AD and PD

There has been an accumulation of evidence regarding the pathological iron accumulation in AD and PD brains [22–24, 29, 30]. Excess iron deposits in the brain can induce oxidative stress, leading to protein aggregation and neuron vulnerability [119, 120]. Furthermore, an increased iron level can lead to up-regulation of translation of APP and α-Syn, 2 key proteins vital to neuron degeneration in AD and PD [48, 51, 63]. Therefore, BBB-penetrating iron chelators should have therapeutic significance for AD and PD patients [50, 116, 121]. The administration of desferrioxamine (DFO), an iron chelator, can slow dementia in AD patients [120]. However, DFO is unstable with poor BBB permeability [122]. Clioquinol (CQ), an iron/Cu/Zn chelator with BBB-penetrating capacity, can clinically alleviate cognitive loss and decrease plasma Aβ levels in AD patients [123]. However, CQ has been identified to be associated with myelinopathies [124]. A second-generation 8-hydroxyquinoline analog metal chaperone, PBT2, was found to be superior to CQ with little adverse events [125, 126]. PBT2 can improve cognition in aged APP C57Bl/6 and Tg2576 transgenic mice, with the effects associated with a decreased interstitial Aβ level [127, 128]. Furthermore, a phase 2 trial with PBT2 in 78 AD patients showed a reduction in Aβ levels in CSF and enhanced performance metrics [125, 126]. Other iron chelators, including VK28 (Fe^3+^ chelator), HLA20 and M30 (Fe^3+^ chelator with N-propargylamine-like properties), can both suppress APP expression and lower the Aβ level in the brain [122]. These results suggest that iron chelators induce down-regulation of APP and Aβ levels via IRP inhibition of APP translation. Iron-chelating agents have also been validated to be effective in PD therapy [129–133]. A natural iron chelator, phytic acid (IP6), has been found to protect DA neurons in an in vitro PD model [129]. Furthermore, iron chelators, including deferasirox, deferiprone, deferrioxamine, VAR10303 and D-607, can all significantly attenuate the loss of DA neurons in various in vivo PD models [130–133]. Detailed future studies are expected to further establish clinical efficacy and safety of iron-chelating strategies in AD and PD therapies.

The intactness of the IRE motif is vital to the functions of the IRP-IRE signaling pathway. Conceivably, point mutations in key nucleotides of the IRE motif of target mRNA can disturb the IRE stem-loop structure and abrogate the interaction between IRPs and the IRE, which can be a pathogenic factor contributing to neuron degeneration in AD and PD. The point mutations in the IRE motif of the H-ferritin gene can lead to inherited human diseases associated with perturbed iron metabolism (iron overload) [92, 134]. Studying whether mutations in the IREs of H-ferritin and other iron metabolism-relevant genes are susceptibility factors for PD and AD will be intriguing. Furthermore, point mutations in the IRE motif of APP and α-Syn genes can be a pathogenic factor for AD and PD. A well-described single nucleotide polymorphism (SNP) associated with AD risk exists in the 5′-UTR of the APP gene [135]. The pathogenic SNP in the 5′-UTR of APP is hypothesized to disturb the IRE motif, leading to increased AD risk [135]. Therefore, more attention should be paid to potential disease-relevant mutations or SNPs in the IRE motifs of the APP and α-Syn genes.

In contrast, point mutations in the coding area of IRP genes, especially the IRP2 gene, may lead to a conformational change of IRP proteins, which can disturb the interaction of IRP proteins with the IRE motifs of APP and α-Syn transcripts. The impaired functional interaction between IRP and the IRE of APP and α-Syn transcripts may up-regulate translation of APP and α-Syn, leading to susceptibility to AD or PD. So far, no mutations in IRPs have been confirmed to be relevant to human disease. However, some SNP polymorphisms in the promoter region of the IRP2 gene have been associated with AD susceptibility [136]. A potentially functional single SNP in the IRP2 promoter region located in the cis-element that interacts with transcription factors may explain the altered IRP2 level in AD patients [136]. Future intriguing findings on these aspects are expected, which should be able to add to our knowledge of the pathogenesis and therapy of AD and PD.

## Conclusions

In summary, recent evidence suggests a pathological and therapeutic link between the iron-IRP-IRE signaling pathway and neuron degeneration in human disorders, especially neurodegenerative diseases. Interesting and significant findings relevant to the pathophysiological roles of the IRP-IRE signaling pathway in human disorders are expected, which can add to our knowledge of the pathogenesis and therapy of comprehensive human disorders, especially incurable human neurodegenerative diseases.

## Abbreviations

AD: Alzheimer’s disease
ADHD: Attention Deficit Hyperactivity Disorder
ALS: amyotrophic lateral sclerosis
APP: amyloid precursor protein
Aβ: amyloid β-peptide
BBB: blood-brain barrier
BCECs: brain capillary endothelial cells
BID: brain iron deficiency
CDC14A: cell division cycle 14 homologue A
COPD: chronic obstructive pulmonary disease
CP: ceruloplasmin
CQ: clioquinol
CSF: cerebrospinal fluid
DA: dopamine
DFO: desferrioxamine
DMT-1: divalent metal transporter 1
DS: Down syndrome
eALAS: erythroid 5-aminolevulinate synthase
eIF4F: eukaryotic initiation factor 4F
FPD: familial form of PD
Fpn: ferroportin
GWAS: genome-wide association study
HHCS: hyperferritinemia-cataract syndrome
HIF: hypoxia-inducible factor
IP6: phytic acid
IRE: iron-responsive element
IRP: iron regulatory protein
JTR-009: APP blocker-9
MCI: mild cognitive impairment
MtFt: mitochondrial ferritin
NAC: N-acetyl-cysteine
NBIA 1: neurodegeneration with brain iron accumulation Type 1
NO: nitric oxide
NP: nature product
PD: Parkinson’s disease
PFTα: pifithrin-α
RLS: Restless Legs Syndrome
ROS: reactive oxygen species
SN: substantia nigra
Tf: transferring
TfR: transferrin receptor
UTR: untranslated region
α-Syn: α-synuclein
